# Radiotherapy for malignant spinal cord compression - prognostic factors for better functional outcome

**DOI:** 10.2478/raon-2026-0013

**Published:** 2026-03-24

**Authors:** Jasna But-Hadzic, Blaz Groselj, Dirk Rades, Barbara Segedin

**Affiliations:** Department of Radiation Oncology, Institute of Oncology Ljubljana, Ljubljana, Slovenia; Faculty of Medicine, University of Ljubljana, Ljubljana, Slovenia; Department of Radiation Oncology, University of Lübeck, Lübeck, Germany

**Keywords:** malignant spinal cord compression, radiotherapy, prognostic factors, outcome

## Abstract

**Background:**

Malignant spinal cord compression (MSCC) is one of the most devastating complications in cancer patients. This retrospective single-center analysis was aimed to identify prognostic factors for better functional outcome after radiotherapy for MSCC.

**Patients and methods:**

Consecutive patients with MSCC treated with upfront radiotherapy between January 2017 and December 2022 were included in this analysis. Data on patient, tumor and treatment characteristics, functional status before and after treatment and diagnostic work-up were collected from the hospital digital database. The treatment was considered effective if performance status (PS) was maintained in PS 1-2 patients or PS improved in PS 3-4 patients.

**Results:**

295 patients were treated for MSCC. The most common primary tumor type was lung cancer (29.3%), followed by prostate (18%) and breast cancer (12%). The treatment was effective in 44.7% of patients. Patients who survived more than 1 month after radiotherapy were more likely to experience functional improvement than patients who died within the first month (60.5% *vs*. 16.5%, p < 0.001). In the multivariate analysis PS 1-2, myeloma/lymphoma, MRI at the time of MSCC and no motor deficits *vs*. paralysis were associated with better functional outcome.

**Conclusions:**

The prognosis of patients with MSCC remains poor. Better stratification of patients to assess possible benefit of radiotherapy for MSCC is warranted.

## Introduction

Malignant spinal cord compression (MSCC) is one of the most distressing complications in cancer patients. It is characterized by worsening pain, progressive neurological deficits resulting in severe quality of life (QoL) deterioration.

The spine is a common site for bone metastases due to its rich vascular supply and lymphatic drainage. The most common mechanism for MSCC is haematogenous spread of malignant cells to the vertebral body, resulting in compression of the spinal artery, venous plexus and spinal cord as the mass grows larger.^1^ MSCC is reported in 5−15% of cancer patients.^1,2^ It most frequently occurs in the thoracic spine, due to its kyphotic shape, exposure to mechanical pressure, narrower spinal canal and larger number of vertebrae. It occurs in all cancer types, with lung, prostate and breast cancer patients accounting for about 60% of all patients with MSCC. In up to 35% of patients MSCC is the first manifestation of the disease, one third of these patients are diagnosed with prostate cancer.^2,3^ In 20 to 34% of cancer patients MSCC is the first systemic manifestation of the disease.^1^ Up to 35% of patients have multiple level MSCC, in 45% of patients more than one vertebra is involved.^1,2,4^

Only early recognition of the signs and symptoms of MSCC, timely clinical assessment and radiological diagnosis allow immediate treatment to prevent irreversible functional damage and deterioration of QoL. Back pain is typically the first symptom of metastases to the vertebrae, reported by up to 95% of patients.^1^ It can appear several months before MSCC, with median reported duration of 6−8 weeks.^2,5-7^ Pain is followed by neurological impairment, that varies according to the spinal location e.g. sensory deficits - paraesthesia, numbness; motor impairment - limb weakness, gait disturbance; and autonomic dysfunction - sphincter dysfunction, loss of sweating below the lesion level, orthostatic hypotension. Autonomic dysfunction and rapid onset MSCC, developing within 24-48 hours and up to 7 days are predictive of worse functional outcome after treatment.^1-3,8^

Radiotherapy with or without surgery is the cornerstone of MSCC treatment. As most patients with MSCC are not eligible for surgery due to the extent of the disease, poor performance status (PS) or poor overall prognosis, the vast majority are treated with upfront radiotherapy.^4^ Numerous different treatment regimens have been reported in the literature.^8-12^ The European Society for Radiotherapy and Oncology - Advisory Committee for Radiation Oncology Practice (ESTRO-ACROP) recommendations, based on the results of four randomized trials and a meta-analysis, recommend 8−10 Gy in a single fraction for all patients with MSCC.^13^ Other authors propose tailoring radiotherapy regimens based on survival prognosis of an individual patient.^14-19^ Rades *et al*. suggest a single fraction treatment as a viable option for patients with a poor overall prognosis, as they achieve comparable results to multiple fraction regimens in pain relief and improvement in motor function.^20-22^ On the other hand, longer course treatments are suggested for patients with an intermediate (5 x 5 Gy) and a good overall prognosis (total dose > 30 Gy).^15^ In field recurrences can be a problem in patients with estimated survival > 12 months after single fraction RT with greater need for re-irradiation.^22,23^ The latest National Institute of Health and Care Excellence (NICE) guideline recommends single 8 Gy fraction treatment for patients with MSCC, with multiple fraction treatment reserved for patients at higher risk of side effects (e.g. large treatment fields, reirradiation).^24^

To effectively personalize treatment, prognostic tools are needed to assess the survival prognosis for each individual patient and to identify patients who would benefit from longer radiotherapy regimens. Several prognostic tools exist, including survival scores and clinical instruments to predict of ambulatory status after radiotherapy.^25,26^ It has been shown that the type of tumor, the functional status of the patient before radiotherapy, the presence of metastases outside the bones and the number of affected organs influence survival, while the tumor type, the ambulatory status before radiotherapy, the presence of sensory deficits and sphincter dysfunction influence the functional outcome after radiotherapy.^25,26^ Yano *et al*. evaluated a magnetic resonance imaging (MRI) grading scale based on the MRI grading system for MSCC developed by Bilsky *et al*., and found that patients with grade 3 spinal cord compression were more likely to be non-ambulatory after radiotherapy.^27,28^

We report the results of a retrospective single-institutional study, aimed to evaluate the functional outcome in patients, treated with upfront radiotherapy for MSCC and to analyse the prognostic factors, affecting functional outcome.

## Patients and methods

This was a retrospective mono-institutional study, approved by the responsible ethics committee (reference number: ERIDEK-0051/2023). We analysed the data of all patients treated with radiotherapy for MSCC at our institution between January 2017 and December 2022. Only patients treated with upfront radiotherapy without prior surgery were included in the analysis. Patients with compression at any spinal level were included in the analysis. In all patients the terminology MSCC is used for the purpose of this analysis, even though in most patients with compression below the second lumbar vertebra cauda equina compression occurs. Electronic medical records were reviewed to extract patient characteristics (gender, age, The Eastern Cooperative Oncology Group [ECOG] PS), disease characteristics (primary tumor type, presence of other bone and/or visceral metastases, number of affected vertebrae, level of MSCC), functional status before radiotherapy (presence of motor deficits, sensory deficits, sphincter dysfunction and pain), diagnostic work-up (neurological examination, MRI, CT, surgical consultation), radiotherapy technique, total dose and treatment outcome. Motor deficits were divided into three categories: no motor deficits, paresis (partial impairment of motor function) and paralysis (complete absence of motor function), whereas sensory deficits, sphincter dysfunction and pain were categorized only as present or absent. A more detailed classification was not possible due to incomplete retrospective data. We calculated the time from diagnosis to MSCC from the date of cancer diagnosis to the documented date of neurological deficits. In patients without neurological deficits the time from diagnosis to the date of MRI showing spinal cord compression served as a surrogate. The interval between confirmed MSCC and the start of radiotherapy was also recorded. Follow-up time was calculated from the start of radiotherapy. Treatment was considered effective if patients with PS 1-2 before radiotherapy maintained the same PS or if their PS improved. For patients with PS 3-4 before radio-therapy, treatment was considered effective only if PS improved after radiotherapy.

### Statistical analysis

Statistical analysis was performed with SPSS® v.26. Descriptive statistics were used to summarize the data, including frequency distributions for categorical variables. The impact of different factors on the efficacy of radiotherapy was assessed in a univariate analysis (UVA) (Chi-square test or Fisher’s exact test when the expected frequency was < 5 for any variable) and multivariate logistic regression analysis. P-values < 0.05 were considered statistically significant and p-values < 0.07 were considered a trend. All variables with p < 0.07 in the univariate analysis were included in the multivariate analysis ([). The Kaplan-Meier method was used to calculate survival time. Univariate (log-rank test) and multivariate Cox regression analyses were used to assess the impact of different factors on survival. All variables with p < 0.07 in the univariate analysis were analysed for independence in a multivariate analysis.

## Results

From January 2017 to December 2022 295 patients were treated with 300 radiotherapy courses for MSCC. In 268 cases MSCC presented with neurological deficits, in 30 cases it presented with pain alone (in these MSCC was described on MRI). In 77 patients (26%) MSCC was the first presentation of an oncologic disease. The most common localization of MSCC was the thoracic spine, followed by the lumbar, cervical and sacral spine. 18.3% of patients had MSCC at multiple levels. The baseline characteristics of the patients, disease and treatment are presented in Table 1.

**TABLE 1.** Patient (N = 295), disease and treatment (N = 300) characteristics

		N (%)
Gender	Female	101 (33.7)
	Male	199 (66.3)
Age (years)	Median (range)	67 (22-91)
ECOG PS	1	24 (8.0)
	2	58 (19.3)
	3	139 (46.3)
	4	79 (26.3)
Primary tumor type	Lung cancer	88 (29.3)
	Prostate cancer	54 (18.0)
	Breast cancer	36 (12.0)
	Urothelial cancer	19 (6.3)
	Multiple myeloma	19 (6.3)
	Lymphoma	20 (6.7)
	Sarcoma	13 (4.3)
	Other^ꟸ^	51 (17.0)
MRI	Yes	211 (70.3)
	No	86 (28.7)
Surgical evaluation	Yes	159 (53.0)
	No	141 (47.0)
Motoric deficit	Paresis	213 (71.0)
	Paralysis	27 (9.0)
	No motoric deficit	60 (20.0)
Pain	Yes	286 (95.3)
	No	14 (5.7)
Sensory deficit	Yes	219 (70.0)
	No	83 (27.7)
Sphincter dysfunction	Yes	97 (32.3)
	No	201 (67.0)
Further bone metastases	Yes	234 (78.0)
	No	65 (21.7)
Visceral metastases	Yes	165 (55.0)
	No	133 (44.3)
Brain metastases	Yes	37 (12.3)
	No	263 (87.7)
Affected spinal level*	Cervical	40
	Thoracic	191
	Lumbar	123
	Sacral	37
Number of affected vertebrae	1-2	192 (64.0)
	3-5	47 (24.7)
	≥ 5	34 (11.3)
Level of MSCC	One level	245 (81.7)
	Multiple levels	55 (18.3)
RT technique	2D	184 (61.3)
	3D CRT	83 (27.7)
	IMRT/VMAT	31 (11.0)
Time to RT in 48h after MSCC onset	No	238 (79.3)
	Yes	62 (20.7)

2D = two dimensional; 3D CRT = three dimensional conformal radiotherapy; ECOG PS = Eastern Cooperative Oncology Group performance status; h = hour; IMRT = intensity modulated radiotherapy; MRI = magnetic resonance imaging; MSCC = malignant spinal cord compression; N = number; RT = radiotherapy; VMAT = volumetric arc therapy

ꟸ Other: Unknown primary (N = 10), colorectal (N = 12), skin (N = 11), head and neck (N = 5), gynecological (N = 2), liver (N = 2), esophageal (N = 2), stomach (N = 5), thyroid (N = 1) and testicular cancer (N = 1). Unknown data is counted as missing value.

* Patients with two (three, four) spinal levels are counted twice (three, four times)

The median time from diagnosis to MSCC was 9 months (range minus 5−373 months). The median time from the onset of MSCC to the start of radiotherapy was 9 days (range 0−145). The median duration of Follow-up time was 2 months (range 0–76 months). Patients were treated with a median of 5 fractions (range 1–12) to a median total dose of 20 Gy (range 8–36 Gy). Improvement of neurological deficits was observed in 32.0% (96), a deterioration in 5.7% (14) and no change in 51.7% (155) of patients. Improvement of any initial PS or maintenance of the same PS in patients with PS 1–2 before radiotherapy was achieved in 44.7% (144). For a summary of the treatment outcomes see Table 2.

**TABLE 2.** Treatment outcomes after radiotherapy for MSCC

		N (%)
Status	Alive	22 (7.3)
	Dead	278 (92.7)
Treatment outcome	No change	155 (51.7)
	Functional improvement	96 (32.0)
	Functional deterioration	17 (5.7)
	Cannot be assessed	32 (10.7)
Improvement in neurological function after RT	No	204 (68%)
	Yes	96 (32%)
Survival > 1 month	No	109 (36.3)
	Yes	190 (63.3)
Effective Treatment*	Yes	134 (44.7)
	No	166 (55.3)

MSCC = malignant spinal cord compression; N = number; RT = radiotherapy

* see definition in Materials and methods section

In the UVA PS, tumor type, MRI for MSCC, presence of motor deficits, sphincter dysfunction, total radiotherapy dose and interval between initial diagnosis and MSCC affected functional outcome in the entire patient cohort. In the MVA only PS 1−2 *vs*. 3−4, myeloma/lymphoma *vs*. other tumor types, MRI at the time of MSCC (yes *vs*. no) and no motor deficits *vs*. paralysis were significantly associated with better functional outcome. Detailed UVA and MVA analyses are presented in Table 3.

**TABLE 3.** Univariate and multivariate logistic regression analysis of prognostic factors affecting functional outcome after radiotherapy for MSCC

Factor	Univariate analysis			Multivariate analysis	
	Subgroup (N)	Effective treatment N (%)	p-value	OR (95% CI)	p-value
ECOG PS	1-2 (82) 3-4 (218)	66 (80.5) 68 (31.2)	**< 0.001**	0.109 (0.054-0.0219)	**< 0.001**
Gender	Female (101) Male (199)	49 (48.5) 85 (42.7)	0.340	Not included in MVA	
Primary tumor type	Lung cancer (88) Prostate cancer (54) Breast cancer (36) **Myeloma/lymphoma (39)** Other (83)	29 (33.0) 25 (46.3) 19 (52.8) 31 (79.5) 30 (36.1)	**< 0.001**	9.257 (3.157-27.146)	NS NS NS **< 0.001** NS
Neurological examination	Yes (159) No (141)	66 (41.5) 68 (48.2	0.234	Not included in MVA	
MRI	Yes (211) No (86)	103 (48.8) 30 (34.9)	**0.029**	2.142 (1.086-4.226)	**0.028**
Surgical evaluation	Yes (159) No (141)	70 (44.0) 64 (45.4)	0.812	Not included in MVA	
Motor deficit	Paresis (213) **Paralysis (27)** No (60)	101 (47.4) 3 (11.1) 30 (50.0)	**0.001**	0.168 (0.086-0.786)	**0.024**
Sensory deficit	Yes (210) No (83)	86 (41.0) 44 (53.0)	0.093	Not included in MVA	
Sphincter dysfunction	Yes (97) No (201)	32 (67.0) 102 (50.7)	**0.004**		NS
Pain	Yes (286) No (14)	129 (45.1) 5 (35,7)	0.490	Not included in MVA	
Further bone metastases	Yes (234) No (65)	105 (44.9) 29 (44.6)	0.667	Not included in MVA	
Visceral metastases	Yes (165) No (133)	71 (43.0) 63 (47.4)	0.335	Not included in MVA	
CNS metastases	Yes (37) No (263)	19 (51.4) 115 (43.7)	0.382	Not included in MVA	
No. of affected vertebrae	1-2 (192) ≥ 3 (108	81 (42.2) 53 (49.1)	0.249	Not included in MVA	
RT technique	2D (184) 3D (83) IMRT/VMAT (31)	74 (40.2) 45 (54.2) 15 (45.5)	0.103	Not included in MVA	
Total RT dose EQD2 (EQD_2_ α/β = 10)	< 31,25Gy (233) ≥ 31,25Gy (67)	95 (40.8) 39 (58.2)	**0.011**		NS
Age (years)	≤ 65 (139) > 65 (161)	60 (43.2) 74 (46.0)	0.627	Not included in MVA	
Time from neurologic deficit to RT (days)	≤ 2 (62) > 2 (238)	22 (35.5) 112 (52.9)	0.102	Not included in MVA	
Time from neurologic deficit to RT (days)	0-7 (132) 8-14 (75) > 14 (93)	57 (43.2) 31 (41.3) 46 (49.5)	0.517	Not included in MVA	
Interval from diagnosis to MSCC (months)	≤ 15 (186) > 15 (114)	74 (39.8) 60 (52.6)	**0.030**		NS

2D = two dimensional; 3D CRT = three dimensional conformal radiotherapy; CI = confidence interval; CNS = central nervous system; ECOG PS = Eastern Cooperative Oncology Group performance status; EQD2 = equivalent dose in 2Gy fractions; IMRT = intensity modulated radiotherapy; MRI = magnetic resonance imaging; MSCC = malignant spinal cord compression; MVA = multivariate analysis; N = number; NS = non significant; OR = odds ratio; RT = radiotherapy; VMAT = volumetric arc therapy

Prognostic factors that retained significance in multivariate logistic regression analysis are shown in bold letters.

In patients with initial PS 3−4 the primary tumor type myeloma/lymphoma was the only prognostic factor that significantly affected functional outcome (OR 10.323, 95% CI 3.417−31.192, p < 0.001). Patients who survived more than 1 month after radiotherapy were more likely to experience functional improvement than patients who died within the first month (60.5% *vs*. 16.5%, p < 0.001).

## Discussion

Radiotherapy is the cornerstone of MSCC treatment as the majority of patients are not candidates for surgical treatment due to the extent of disease, poor PS or overall prognosis. New prognostic factors and tools are needed to identify those patients who would benefit from different fractionation regimens or only from best supportive care.

We present the results of one of the largest mono-institutional retrospective series of patients treated with radiotherapy for MSCC. We evaluated the effect of treatment in terms of functional outcome and prognostic factors for better functional outcome to compare with previously published clinical trials.

In line with other studies, we included patients with compression at any level of the spine in our analysis.^11,29^ In cauda equina compression the degree of urgency is sometimes considered to be lower than in other levels of the spine and the response to radiotherapy different. In a secondary analysis of the SCORAD study the anatomical level of compression was included as a prognostic factor. Their multivariable models and nomogram used a binary split (C1–T12 *vs*. L1–S5) and found that compression at C1–T12 was associated with worse overall survival at 8 weeks after radiotherapy. No effect on the functional outcome was reported.^20^ Lee *et al*. reported no effect of compression level on the functional outcome, Maranzano *et al*. did not perform a separate analysis.^16,19^ Additionally, none of the scoring tools include the level of compression as a prognostic factor.

Treatment with radiotherapy was defined as effective if patients with an initial ECOG PS 1-2 had the same or better PS after radiotherapy. In patients with PS 3-4 before radiotherapy, treatment was considered effective only if PS improved. Our definition is in agreement with the definition by Rades *et al*., who defined treatment as effective in patients with improved motor function or remaining ambulatory without aid.^25^ Radiotherapy was effective in 44.7% of patients in our cohort, 36% of patients in the cohort died within 4 weeks after radiotherapy, in this group, treatment was effective in only 16.5% of patients. In patients who survived longer than 4 weeks, treatment was effective in 60.5% (p < 0.001). Randomized studies by Hoskin *et al*. and Maranzano *et al*. report better functional outcomes in both treatment arms, compared to our retrospective data in the whole cohort, while our functional outcomes in the subgroup of patients, who survived more than one month are more in line with the functional outcomes reported in the SCORAD study.^14,20,29^ In the primary analysis of SCORAD study, only patients surviving more than two months were included and the ambulatory status grade 1 or 2 was present in 69.3% and 72.7% in the single fraction and multi-fraction group, respectively.^29^ In a large meta-analysis, walking ability was present in 49% of patients before and 69.7% of patients after treatment of MSCC and 87% of patients maintained ambulation.^4^ Patients treated with different types of surgery with or without postoperative radiotherapy as well as patients treated with up-front radiotherapy were included in the analysis.

We identified four independent predictors of functional outcome. PS 1−2 and myeloma/lymphoma tumor type were associated with better functional outcome after radiotherapy, whereas the presence of paralysis was associated with a worse functional outcome compared to no motor deficits. Similar to our results, Rades *et al*. reported that tumor type (breast cancer and myeloma/lymphoma) and ambulatory status before radiotherapy were independent predictors of functional outcome. In addition, sensory deficits and sphincter dysfunction affected functional outcome and are included in a clinical instrument, predicting ambulatory status after radiotherapy.^25^ In our study, the presence of sensory deficits and sphincter dysfunction were not identified as prognostic factors for functional outcome. Rades *et al*. developed the first prognostic tool for ambulatory status after radiotherapy for MSCC in 2008.^30^ In 2022 a new prognostic tool was developed, that proved to be more accurate in predicting ambulatory status after radiotherapy.^25^ We validated the latest clinical instrument, published by Rades *et al*. on our data set to stratify our patients into prognostic groups estimating post-treatment ambulatory rate after radiotherapy. Post-radiotherapy ambulatory rates for our patients were 23.2%, 41.8% and 85.7% for those stratified into prognostic groups I, II and II, respectively. In the original paper by Rades *et al*., post-radiotherapy ambulatory rates were 10%, 65% and 97% in the corresponding prognostic groups. We found a significant difference in the ambulatory rates in groups I and II, compared to the cohort from the original paper. Compared to the new prognostic score, the results in our cohort are better in patients, stratified in the group with the worst functional prognosis (group I) and worse in patients, stratified in groups with a better functional prognosis (groups II and III) and overall. The prospective data used to develop the prognostic tool included a prospective study in which all patients were treated with 5x5 Gy and a prospective study that included patients with a favorable OS prognosis who were treated with longer course radiotherapy with higher total doses. These facts may limit the generalizability of the score to more heterogeneous groups of patients with MSCC. Nevertheless, using this prognostic tool, our patients were stratified into three prognostic groups with different rates of ambulatory status after radiotherapy. Due to the retrospective nature of our analysis a detailed classification of ambulatory status was not possible, which is why we used the patient’s PS as a surrogate. The assessment of PS is very subjective. Many of the patients with MSCC included in the analysis were treated urgently during night or weekend shifts, when the radiation oncologist covers multiple working stations, which could lead to a bias in PS assessment due to lack of time. In addition, many physicians were involved in the clinical treatment of the patients, which increases the inhomogeneity of the data. In cases where PS was not assessed as a numerical value (0 to 4) by the treating physician, we assessed PS at the time of data collection based on clinical description and neurological status, where it was available. This led to additional possible bias in our analysis. Regardless of the differences in ambulatory status rates, the prognostic tool seems to work well and should be considered when deciding on a personalized treatment approach in patients with MSCC.

Interestingly, MRI at the time of MSCC was also significantly associated with a better functional outcome in our study. To our knowledge, this is the first study to show this result. One possible reason for this could be, that we miss multiple level MSCC in patients without pretreatment MRI. On the other hand, a proportion of our patients had an MRI of the affected spinal level only, which could also be a reason for undetected multiple level MSCC. MRI is always performed in patients with good PS, while clinicians sometimes chose to do a CT scan in patients with poor PS and/or poor prognosis. However, both PS and MRI were independent predictors of functional outcome in MVA. Reports on the value of MRI in MSCC are sparse. A randomized study by Dearnaley *et al*. reports on the screening effect of MRI in detecting asymptomatic MSCC in patients with castration resistant prostate cancer with spinal metastases. They found neither a survival benefit nor a difference in the incidence of MSCC by MRI screening.^31^ The effect of MRI on the functional outcome of radiotherapy for MSCC should therefore be confirmed in a prospective study.

Better assessment of patients who would benefit from radiotherapy for MSCC is warranted. Rades *et al*. developed a score to identify patients near the end of life, who would benefit from radiotherapy for MSCC.^32^ In their retrospective analysis, pretreatment ambulatory status, myeloma/lymphoma tumor type and motor deficits that have been developing for more than 14 days were associated with both postradiotherapy ambulatory status and effective treatment in patients who died within 2 months of radiotherapy.^11,32^ Giraldo *et al*. reported a moderate pain response 2 weeks after radiotherapy with insignificant effects on motor and sphincter function in patients with short life expectancy.^11^ In our group of patients, who died within 1 month after radiotherapy, only 18 (18.3%) patients were PS 1-2 (ambulatory) before radiotherapy. Considering our results in an unselected group of patients and the results reported by Giraldo *et al*. and Rades *et al*., radiotherapy for MSCC near the end of life seems to be justified in patients with a better estimated functional out-come and in selected patients with poor response to analgesic treatment.

In our study, the total RT dose in EQD_2_ had no effect on functional outcome. The meta-analysis by Liu *et al*., which included 4897 MSCC patients with various primary tumors and in a review by da Silva *et al*., which only included MSCC patients with malignant melanoma, also showed no effect of the total dose on the functional outcome.^4,33^ Several randomized studies showed no advantage of multiple-fraction radiotherapy compared to single-fraction radiotherapy in MSCC.^16,17,19,29^ Rades *et al*. reported that fractionated therapies (> 30 Gy) improve local control in long-term survivors, but single-fraction RT is preferable in patients with poor prognosis.^15^ While the ESTRO ACROP guidelines recommend single fraction radiotherapy for patients with MSCC based on the results of these randomized studies, we believe that stratification of patients by survival prognosis and personalization of radiotherapy is worth considering.

Our study has some limitations. Patients from a single center were included and analyzed and the study is retrospective. Therefore, most of the data in our cohort study had to be extracted from the patients’ medical records, which were often incomplete. As pain was not quantitatively assessed at follow-up, we were unable to assess the pain response to radiotherapy. Also, no quality of live (QoL) questionnaires were routinely given to the patients before radiotherapy for MSCC, so assessment of radiotherapy effect on QoL was not possible. Furthermore, we did not include data on systemic treatment in the analysis, although some studies report better survival in patients with MSCC, who also received systemic treatment, but this has not been further validated in any scoring tools.^34^ We believe the role of systemic therapy has to be considered for long-term disease control based on tumor histology and systemic disease status, but its role in improving immediate neurologic recovery or ambulatory function in combination with radiotherapy has not been established in prospective or retrospective studies. In our cohort of patients with poor survival the potential role of systemic therapy is very limited. Because our cohort is large, many clinicians were involved in decision making both regarding diagnosis and treatment, making it a very heterogenous group. The radiotherapy dose and fractionation schedules were at the discretion of each treating physician, so many different schedules were used. Patients with PS 3−4 were more likely to receive a lower total dose (< 31.25 Gy) than patients with PS 1−2.

## Conclusions

A modern treatment approach for MSCC should be rapid and individualized. The tumor type and PS/ambulatory status should be taken into account when determining the optimal treatment plan. MRI of the entire spine should be routinely used to detect the level(s) of MSCC.

In this study, we identified some prognostic factors for optimal functional and survival outcome in MSCC patients treated with radiotherapy. Survival remains poor in this patient population, with better outcomes in patients with myeloma/lymphoma and breast cancer. Radiotherapy has been shown to be ineffective in patients at/near the end of life. For most of these patients, best supportive care is likely to be the optimal treatment option. A selected group of patients, who would benefit from radiotherapy can be identified using already available scoring tools. In order to achieve better treatment outcomes and avoid unnecessary treatment, more careful patient selection is required. The prognostic factors should be further tested in prospective clinical trials.
